# When the cardiac magnetic resonance rescues the surgeon: an unusual clinical case of a right atrial myxoma and a post-operative pseudomass

**DOI:** 10.1093/ehjcr/ytae022

**Published:** 2024-01-17

**Authors:** Mariana Sousa Paiva, Rita Reis Santos, Sara Guerreiro

**Affiliations:** Centro Hospitalar de Lisboa Ocidental, Hospital de Santa Cruz, Mariana Sousa Paiva, Av. Prof. Dr. Reinaldo dos Santos, Carnaxide 2790-134, Portugal; Centro Hospitalar de Lisboa Ocidental, Hospital de Santa Cruz, Mariana Sousa Paiva, Av. Prof. Dr. Reinaldo dos Santos, Carnaxide 2790-134, Portugal; Centro Hospitalar de Lisboa Ocidental, Hospital de Santa Cruz, Mariana Sousa Paiva, Av. Prof. Dr. Reinaldo dos Santos, Carnaxide 2790-134, Portugal

**Keywords:** Cardiac mass, Transthoracic echocardiography, Cardiac magnetic resonance, Multi-modality imaging

A 72-year-old woman with a past medical history of hypertension and dyslipidaemia was referred to cardiac surgery consultation because of an asymptomatic right atrial mass detected on a routine transthoracic echocardiogram (TTE) (*Figure 1A*, Supplementary material online, *Videos S1, S2* and *S4*).

**Figure 1:**
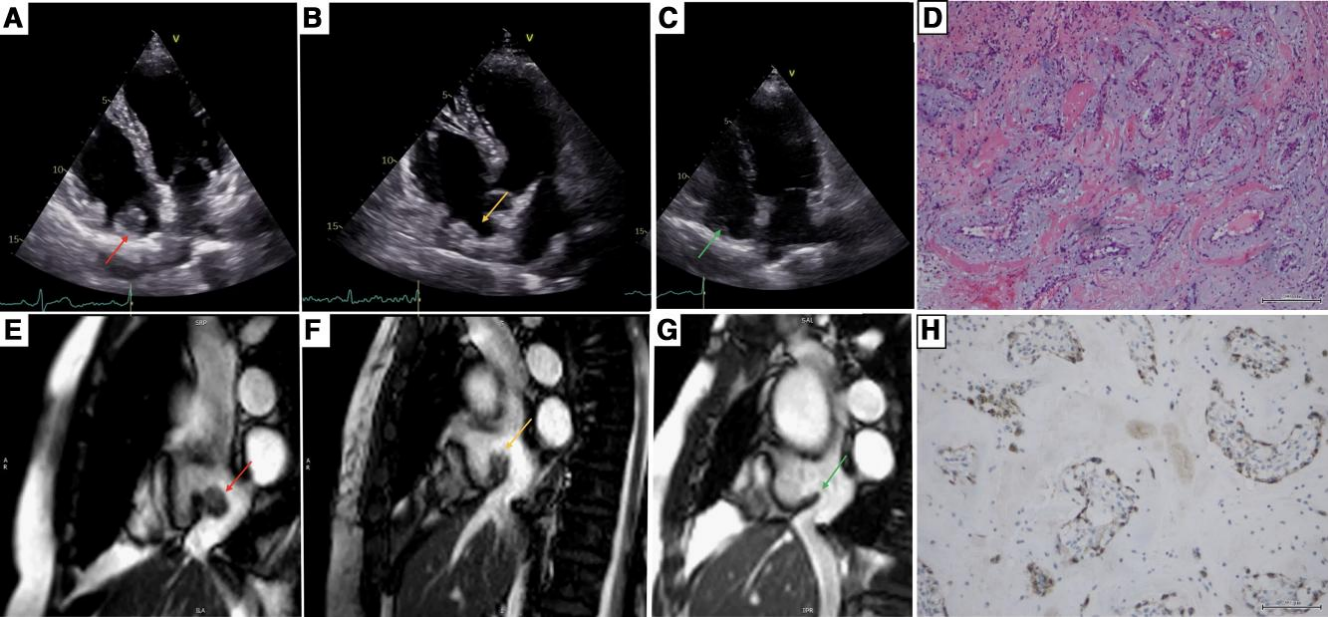
Transthoracic echocardiogram: (*A*) right ventricle-focused four-chamber view showing an ovoid mass (red arrow) at the right atrium before surgery; (*B*) right ventricle-focused four-chamber view depicting a similar mass (orange arrow) at the same location at 1-week follow-up; and (*C*) right ventricle-focused four-chamber view exhibiting the crista terminalis without any attached mass (green arrow) at 3-month follow-up. Cardiac magnetic resonance: (*E*) cine steady-state free precession (SSFP) at bicaval view showing the mass (red arrow) connected to the crista terminalis before surgery; (*F*) cine SSFP at bicaval view revealing the crista terminalis (orange arrow) with increased size compared with the previous study; (*G*) cine SSFP at bicaval view depicting the crista terminalis (green arrow) without any attached mass. Histopathology: (*D*) (haematoxylin and eosin)—myxoma composed of round to oval and stellate cells in abundant loose myxoid matrix containing abundant mucopolysaccharides; (*H*) (immunohistochemistry study)—calretinin immunoreactivity is shown by myxoma cells that are arranged around vessels or isolated and scattered within the myxoid stroma.

Initial physical examination, laboratory tests, and electrocardiogram revealed no abnormal findings. A cardiac magnetic resonance (CMR) (*Figure 1E*) confirmed the presence of an ovoid mass (red arrow) with regular borders arising from crista terminalis and showed heterogeneous late gadolinium enhancement (LGE). Overall, the imaging findings were consistent with a cardiac tumour, namely a myxoma.

As myxomas carry a risk of embolization, the mass (15 × 14 × 11 mm) was completely resected without further complications. Nevertheless, the post-operative TTE study unexpectedly revealed a similar mass at the same location (*Figure 1B*). To confirm the complete resection, a new CMR was performed showing enlarged and irregular crista terminalis, isointense in T1 and T2, without LGE and without any mass attached (*Figure 1F*). Histopathological examination confirmed the diagnosis of myxoma (*Figure 1D* and *H*). At 3-month follow-up, the patient remained asymptomatic, and the imaging study revealed the complete disappearance of the pseudomass (*Figure 1C* and *G,*Supplementary material online, *Video S3*). These findings are thought to be linked with surgical erosion of the atrial wall and possibly crista terminalis haematoma.

Myxomas are the most common primary cardiac tumours and can arise from unusual locations. Cardiac surgery and healing may distort the structure of tissues creating artefacts in post-operative imaging that require careful interpretation. Multi-modality imaging plays an important role in cardiac mass evaluation and CMR is a crucial tool for differential diagnosis as it allows detailed tissue characterization.

## Supplementary Material

ytae022_Supplementary_Data

## Data Availability

No new data were generated or analysed in support of this research.

## Notes

**Consent:** Written informed consent from the patient was obtained and archived and it is available.

**Funding:** This research did not receive any specific grant from funding agencies in the public, commercial, or not-for-profit sectors.

**Ethics approval:** The authors confirm that this report is in accordance with internationally accepted recommendations for clinical investigation, namely the Declaration of Helsinki of the World Medical Association.

