# Supplementary material for: When the cardiac magnetic resonance rescues the surgeon: an unusual clinical case of a right atrial myxoma and a post-operative pseudomass
Source: Eur Heart J Case Rep. 2024 Jan 17;8(1):ytae022. doi: 10.1093/ehjcr/ytae022 (PMC10810044; doi:10.1093/ehjcr/ytae022)
Supplement: ytae022_Supplementary_Data [file ytae022_supplementary_data.zip › Supplementary materials.docx]

**Supplementary materials:**

**Supplementary video 1:** Bi-plane video via three-dimensional (3D) transthoracic echocardiography (reference view: right ventricle focused 4-chamber) with Color Doppler evaluation of the mass before surgery.

**Supplementary video 2:** Bi-plane video via three-dimensional (3D) transthoracic echocardiography (reference view: right ventricle focused 4-chamber) of the mass before surgery.

**Supplementary video 3:** Bi-plane video via three-dimensional (3D) transthoracic echocardiography (reference view: right ventricle focused 4-chamber) showing *crista terminalis* at 3-month follow-up.

**Supplementary video 4:** Right atrial mass via real-time three-dimensional (3D) transthoracic echocardiography.
